# ^11^C-DPA-713 Versus ^18^F-GE-180: A Preclinical Comparison of Translocator Protein 18 kDa PET Tracers to Visualize Acute and Chronic Neuroinflammation in a Mouse Model of Ischemic Stroke

**DOI:** 10.2967/jnumed.118.209155

**Published:** 2019-01

**Authors:** Aisling Chaney, Haley C. Cropper, Emily M. Johnson, Kendra J. Lechtenberg, Todd C. Peterson, Marc Y. Stevens, Marion S. Buckwalter, Michelle L. James

**Affiliations:** 1 Department of Radiology, Stanford University, Stanford California; 2 Department of Neurology and Neurological Sciences, Stanford University, Stanford California; and; 3 Department of Neurosurgery, Stanford University School of Medicine, Stanford, California

**Keywords:** TSPO, ischemic stroke, neuroinflammation, PET

## Abstract

Neuroinflammation plays a key role in neuronal injury after ischemic stroke. PET imaging of translocator protein 18 kDa (TSPO) permits longitudinal, noninvasive visualization of neuroinflammation in both preclinical and clinical settings. Many TSPO tracers have been developed, however, it is unclear which tracer is the most sensitive and accurate for monitoring the in vivo spatiotemporal dynamics of neuroinflammation across applications. Hence, there is a need for head-to-head comparisons of promising TSPO PET tracers across different disease states. Accordingly, the aim of this study was to directly compare 2 promising second-generation TSPO tracers, ^11^C-DPA-713 and ^18^F-GE-180, for the first time at acute and chronic time points after ischemic stroke. **Methods:** After distal middle cerebral artery occlusion or sham surgery, mice underwent consecutive PET/CT imaging with ^11^C-DPA-713 and ^18^F-GE-180 at 2, 6, and 28 d after stroke. T2-weighted MR images were acquired to enable delineation of ipsilateral (infarct) and contralateral brain regions of interest (ROIs). PET/CT images were analyzed by calculating percentage injected dose per gram in MR-guided ROIs. SUV ratios were determined using the contralateral thalamus (SUV_Th_) as a pseudoreference region. Ex vivo autoradiography and immunohistochemistry were performed to verify in vivo findings. **Results:** Significantly increased tracer uptake was observed in the ipsilateral compared with contralateral ROI (SUV_Th_, 50–60 min summed data) at acute and chronic time points using ^11^C-DPA-713 and ^18^F-GE-180. Ex vivo autoradiography confirmed in vivo findings demonstrating increased TSPO tracer uptake in infarcted versus contralateral brain tissue. Importantly, a significant correlation was identified between microglial/macrophage activation (cluster of differentiation 68 immunostaining) and ^11^C-DPA-713- PET signal, which was not evident with ^18^F-GE-180. No significant correlations were observed between TSPO PET and activated astrocytes (glial fibrillary acidic protein immunostaining). **Conclusion:** ^11^C-DPA-713 and ^18^F-GE-180 PET enable detection of neuroinflammation at acute and chronic time points after cerebral ischemia in mice. ^11^C-DPA-713 PET reflects the extent of microglial activation in infarcted distal middle cerebral artery occlusion mouse brain tissue more accurately than ^18^F-GE-180 and appears to be slightly more sensitive. These results highlight the potential of ^11^C-DPA-713 for tracking microglial activation in vivo after stroke and warrant further investigation in both preclinical and clinical settings.

Neuroinflammation is a potent driver of neuronal damage and degeneration after ischemic stroke (1,2). Activation of glia and infiltration of peripheral immune cells into the brain are central to both the detrimental consequences observed in acute phases after stroke and the neuroprotective effects, contributing to neuronal repair, survival, and damage limitation (2). Although the connection between neuroinflammation and ischemic stroke is unrefuted, the in vivo spatiotemporal dynamics of specific immune cells, at acute and chronic time points, in individual stroke patients is poorly understood. Moreover, how these immune signatures relate to clinical outcomes remains unknown.

Investigating the multifaceted molecular aspects of the innate and adaptive immune response in the central nervous system after stroke is mostly restricted to in vitro postmortem analyses (e.g., immunologic assays). Although these techniques continue to provide invaluable insights into the complex neuroimmune interactions after ischemia, they are limited to a single time point of inquiry and thus cannot provide in vivo longitudinal data needed to elucidate this dynamic process. With increasing evidence linking chronic neuroinflammation to depression, fatigue, and cognitive decline after stroke (3–5), there is a growing need to accurately quantify neuroinflammation in vivo. Currently, there are no routine in vivo methods approved for detecting and monitoring the innate or adaptive immune cells noninvasively. Therefore, there is a critical need for specific molecular imaging biomarkers to enhance our understanding of the immune response in acute and chronic phases after ischemia. Such imaging biomarkers would afford unique insights into an individual patient’s immune signature and help predict clinical outcomes, including risk of poststroke dementia (6,7). Furthermore, in vivo tracking of neuroinflammation after stroke could provide a means to select patients for novel immune-targeted therapeutics, identify appropriate time windows for meaningful intervention, and monitor treatment response, thus expediting development and translation of efficacious therapies.

The translocator protein 18 kDa (TSPO) represents such a biomarker, for which numerous PET radiotracers have been developed. TSPO expression is high in peripheral tissues, including kidneys, lungs, and steroid-associated tissues (e.g., adrenal glands), and low in healthy brain tissue, where it is mainly restricted to microglia, and to a lesser extent astrocytes (8,9). On injurious proinflammatory stimulation, TSPO protein levels markedly increase in activated microglia and infiltrating myeloid cells, providing a valuable imaging biomarker of activated innate immune cells and neuroinflammation (8,10). The first TSPO PET tracer to be widely evaluated for imaging neuroinflammation was ^11^C-PK11195. Although ^11^C-PK11195 provided an opportunity to visualize neuroinflammation in living subjects for the first time, it is unfortunately limited by inadequate brain penetration and high nonspecific binding, resulting in low signal-to-background and poor sensitivity (11). Numerous second-generation TSPO PET tracers have been developed to improve these limitations, including ^11^C-PBR28 (12), ^11^C-DPA-713 (13), ^18^F-DPA-714 (14), ^18^F-PBR06 (15), ^18^F-FEPPA (16), ^11^C-DAA1106 (17), and ^18^F-GE-180 (18). Although many have shown increased sensitivity and affinity compared with ^11^C-PK11195 (19–22), no head-to-head studies have been conducted using 2 second-generation tracers in the context of stroke. Here, we chose 2 promising second-generation tracers, ^11^C-DPA-713 and ^18^F-GE-180 (both reported to have higher sensitivities than ^11^C-PK11195), and directly compared their sensitivity and accuracy for detecting acute and chronic neuroinflammation in the distal middle cerebral artery occlusion (dMCAO) mouse model of stroke. As a secondary aim, we investigated the utility of TSPO PET for quantifying alterations in peripheral inflammatory responses in the spleen.

## MATERIALS AND METHODS

### Study Design

**FIGURE 1.**
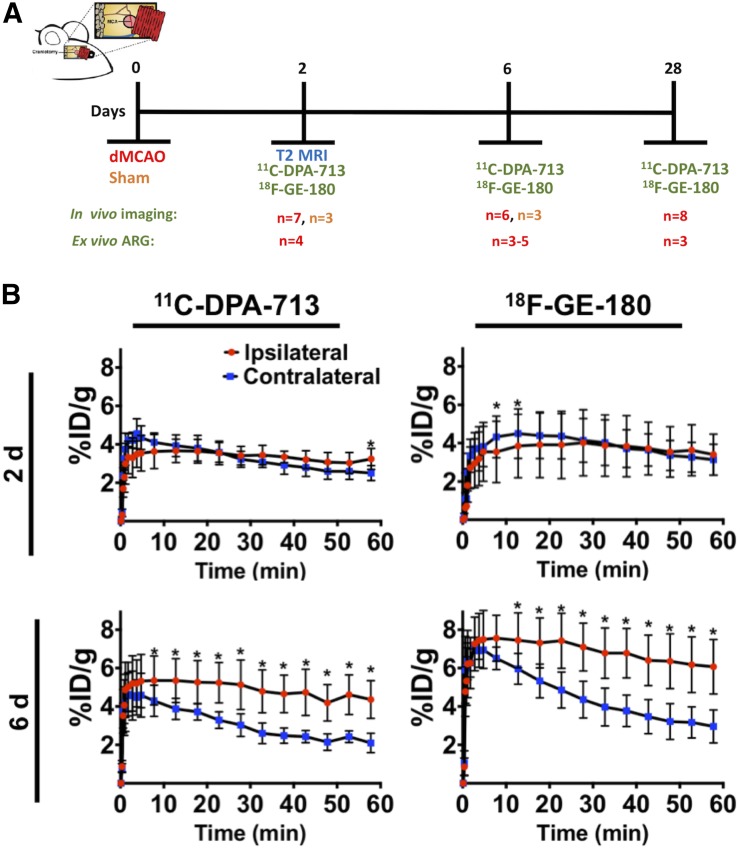
(A) Study design timeline adapted from Doyle et al. (23). (B) Time–activity curves depicting dynamic ^11^C-DPA-713 and ^18^F-GE180 uptake (%ID/g) in ipsilateral and contralateral brain ROIs at 2 and 6 d after dMCAO surgery (mean ± SD). Wilcoxon matched paired test (*P < 0.05).

The dMCAO mouse model of stroke was chosen for this study because of the reproducible, restricted ischemic damage and low mortality rates associated with this surgery (23,24). Longitudinal TSPO PET imaging of dMCAO mice has yet to be reported; however, elevated TSPO PET signal peaks between 3 and 11 d after stroke using other rodent models of ischemia (e.g., MCAO) (25–28). Consequently, a 6-d time point was chosen to ensure the presence of elevated TSPO levels. Additionally, 2- and 28-d time points were selected to determine whether TSPO PET could be used to detect acute and chronic inflammation known to occur in this model (Fig. 1A) (3). MRI was performed 2 d after dMCAO, to provide confirmation of stroke and an anatomic reference for PET image analysis, and was followed by sequential in vivo ^11^C-DPA-713 and ^18^F-GE-180 PET imaging. For all time points, ^11^C-DPA-713 PET was performed first, followed by ^18^F-GE-180 PET after sufficient radioactive decay of ^11^C (i.e., 10 half-lives). After TSPO PET, brain tissues were collected to perform immunohistochemistry (*n* = 3–5) to investigate the relationship between TSPO PET signal for each tracer and glial activation. Additionally, ex vivo autoradiography (*n* = 3–5) was performed for each tracer to obtain high-spatial-resolution images to confirm in vivo findings. A small cohort of mice underwent sham surgery (*n* = 3) to control for possible inflammatory responses caused by surgery alone and were imaged at either 2 or 6 d.

### dMCAO Surgery

Protocols approved by Stanford University’s Institutional Animal Care and Use Committee were used for all animal experiments. Surgery via craniotomy and permanent dMCAO was performed as previously outlined on 3-mo-old female C57BL/6J mice (23). Sham surgery involving craniotomy and manipulation of the meninges (without dMCAO) was also performed. After surgery, animals were administered subcutaneous cefazolin, 25 mg/kg (VWR #89149-888) and buprenorphine SR, 1 mg/kg (Zoopharm) and were monitored until fully ambulatory.

### Radiosynthesis

Radiosynthesis of ^11^C-DPA-713 and ^18^F-GE-180 was completed according to standard methods (13,18) and formulated in phosphate-buffered saline (0.1 mol/L NaCl, 0.05 mol/L sodium phosphate, pH 7.4) containing 10% ethanol. Both tracers were obtained with high specific radioactivity (^11^C-DPA-713: 198.9 ± 10.8; ^18^F-GE-180: 128.2 ± 13.1 GBq/μmol) and radiochemical purity (>99%) (*n* = 4).

### MR Imaging

Three-dimensional axial (coronal) T2-weighted MR images (echo time: 33 ms; retention time: 2,500 ms; 2 averages; 17 slices) were acquired using a millipede quadrature radiofrequency coil on a 7-T MRI Varian Magnex Scientific MR scanner system as previously reported (29).

### PET Imaging

Dynamic PET images 2 and 6 d after dMCAO surgery were acquired over 60 min using a dual microPET/CT scanner (Inveon; Siemens) as previously described (29). Static imaging (10 min) was conducted at 50 min after tracer injection for the 28-d time point. Each mouse was intravenously injected via tail vein with 7–11.5 MBq of ^11^C-DPA-713. After a minimum of 10 half-lives, the same mice were anesthetized and injected with 5.4–11.8 MBq of ^18^F-GE-180. Additional blocking studies were conducted at 6-d after dMCAO. PK11195 (3 mg/kg) was administered intravenously 15 min before tracer injection, and dynamic 60-min PET acquisition was performed.

### Image Analysis

Images were analyzed using VivoQuant software (version 3.0; inviCRO) as previously described (29). In brief, PET, CT, and MR images were coregistered, and MR-guided ROIs were manually drawn for infarcted/ipsilateral and contralateral tissue (Supplemental Fig. 1A; supplemental materials are available at http://jnm.snmjournals.org). To permit accurate quantification of PET tracer uptake without using an invasive arterial input function, a suitable internal reference region is required. This region should have low (if any) specific tracer uptake, and the signal should not differ between study groups or areas of interest. Although TSPO levels are low in the healthy brain, no region is truly devoid of TSPO expression, hence a brain reference region should be referred to as a pseudoreference region. Here, a split-brain atlas was used to quantify tracer uptake in brain structures in left versus right hemispheres in an unbiased manner, which revealed the contralateral thalamus as a pseudoreference region due to its low TSPO PET signal that did not vary from uptake in the ipsilateral thalamus (Supplemental Figs. 1B and 1C). SUV ratios using the contralateral thalamus (SUV_Th_) were calculated by dividing the ROI (i.e., infarct or contralateral) uptake by that of the contralateral thalamus. Because clinical TSPO PET stroke studies use the ipsilateral cerebellum as a reference region (30), the suitability of this structure as a clinically relevant reference region was also assessed (by calculating SUV_Cb_ ratios) (Supplemental Fig. 2). Tracer uptake in the spleen was also quantified to assess peripheral inflammation using both the CT and the PET images for guidance, ensuring no overlap with kidney uptake. Inveon Research Workstation was used for PET image visualization.

### Autoradiography

Ex vivo autoradiography was performed using previously reported methods (29). Briefly, 20-μm-thick brain sections were collected 30 min after injection of 26.6–71.8 MBq of ^11^C-DPA-713 and 50 min after injection of 23–40.5 MBq of ^18^F-GE-180 at all time points after dMCAO. After exposing tissues to digital autoradiography films for 10 half-lives, each film was scanned using a typhoon phosphorimager. ImageJ software version 2.0.0 was used to quantify ipsilateral–to–contralateral uptake ratios to account for any differences in radioactivity injected between tracers.

### Immunohistochemistry Staining and Quantitation

For semiquantitative evaluation of microgliosis and astrogliosis, cluster of differentiation 68 (CD68) and glial fibrillary acidic protein (GFAP) staining were performed, respectively, using previously described methods (31). Images were captured in the infarct border and contralateral cortex at 20× via a Nanozoomer 2.0-RS (Hamamatsu) using 5 sections spaced 480 μm apart per mouse. Masked, unbiased quantification of the area covered by staining in these images was performed using ImageJ software. Immunofluorescent TSPO/CD68 and TSPO/GFAP double staining was performed, as previously described (32), on dMCAO mice 6-d tissue to assess the extent of TSPO-positive microglia versus astrocytes underlying the TSPO PET signal.

### Statistics

GraphPad Prism (version 7; GraphPad Software) was used for statistical analyses of the data using *t* tests, 1-way ANOVA, and 2-way ANOVAs with multiple comparisons. A *P* value of 0.05 or less was considered significant.

## RESULTS

To directly assess the sensitivity of ^11^C-DPA-713 versus ^18^F-GE-180, a head-to-head comparison was conducted via sequential PET imaging of the same mice at acute (2 and 6 d) or chronic (28 d) time points after stroke. Time–activity curves at 2 d after dMCAO demonstrated small differences in ipsilateral compared with contralateral brain ROI uptake (percentage injected dose per gram [%ID/g]) with significantly higher uptake seen at 55–60 min, with ^11^C-DPA-713 but not with ^18^F-GE-180 (Fig. 1B). At 6 d, there was markedly increased uptake in the ipsilateral compared with the contralateral ROI for both tracers. Since the highest signal-to-background ratios were observed at 50–60 min after injection, this time point was chosen for quantification and subsequent static acquisitions at 28 d.

**FIGURE 2.**
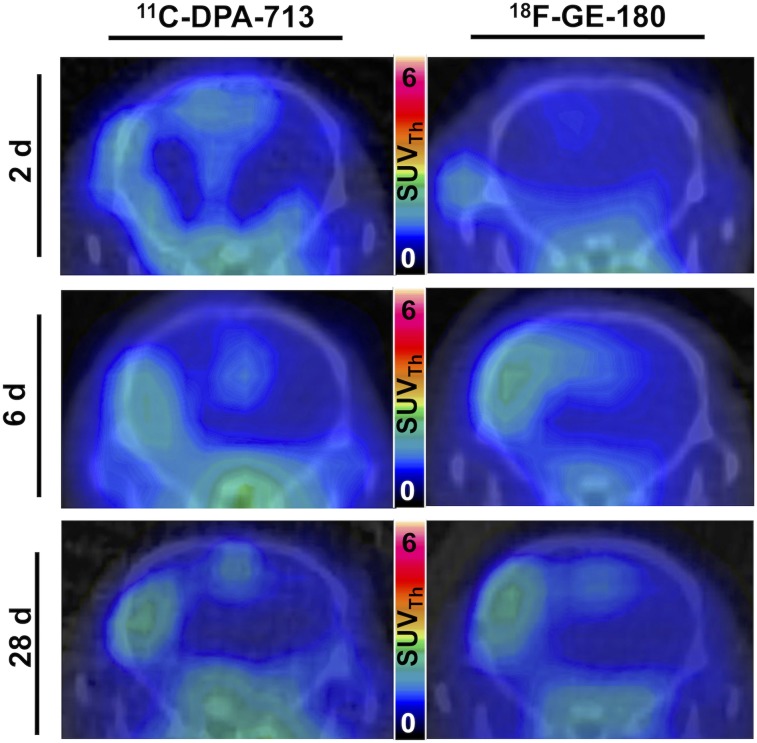
Representative ^11^C-DPA-713 and ^18^F-GE180 PET/CT coronal mouse brain images of dMCAO mice (SUV_Th_).

**FIGURE 3.**
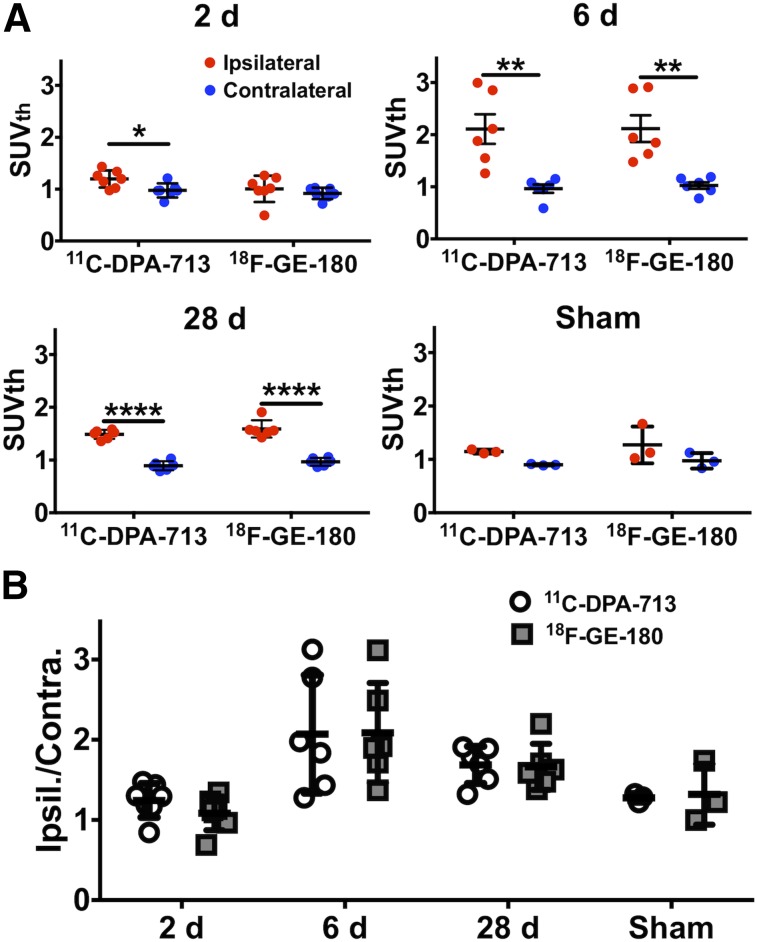
(A) PET image quantification of ^11^C-DPA-713 and ^18^F-GE-180 uptake (SUV_Th_) in ipsilateral and contralateral ROIs in dMCAO mice at 2, 6, and 28 d and sham mice at 6 d after surgery (mean ± SD). (B) Ipsilateral to contralateral uptake ratios in dMCAO and sham mice (mean ± SD). Two-way ANOVA, Sidak’s post hoc test (*P < 0.05, **P < 0.01, ****P < 0.0001).

Summed 50- to 60-min PET/CT images showed increased tracer uptake in the ipsilateral hemisphere at both acute and chronic time points (Fig. 2). Quantification revealed a significant increase in tracer uptake (SUV_Th_) in the ipsilateral compared with the contralateral ROI at 2 d after dMCAO using ^11^C-DPA-713 (1.20 ± 0.06 vs. 0.98 ± 0.05, *P* < 0.05, *n* = 7) but not ^18^F-GE-180 (1.01 ± 0.96 vs. 0.92 ± 0.42, *P* > 0.50, *n* = 7) (Fig. 3A). Significantly increased ipsilateral uptake was observed for ^11^C-DPA-713 and ^18^F-GE-180 at 6 d (^11^C-DPA-713: 2.11 ± 0.29 vs. 0.96 ± 0.08, *P* < 0.01; ^18^F-GE-180: 2.12 ± 0.26 vs. 1.03 ± 0.06, *P* < 0.01). Increased ipsilateral uptake was maintained for both tracers at 28 d after dMCAO (^11^C-DPA-713: 1.49 ± 0.04 vs. 0.89 ± 0.04, *P* < 0.0001; ^18^F-GE-180: 1.59 ± 0.07 vs. 0.97 ± 0.03, *P* < 0.0001). Conversely, low brain uptake was observed for sham mice (2 and 6 d after surgery, Supplemental Fig. 4 and Fig. 3A, respectively), with no significant differences between ipsilateral and contralateral ROI uptake observed for either tracer. The ratio of ipsilateral-to-contralateral uptake at 2, 6, and 28 d after dMCAO did not differ significantly between tracers (Fig. 3B). Similar findings were observed when using the ipsilateral cerebellum as a pseudoreference region (SUV_Cb_) (Supplemental Fig. 2). Blocking with PK11195 at 6 d after dMCAO revealed a significant decrease in TSPO PET signal in the infarct ROI of dMCAO mice using both ^11^C-DPA-713 and ^18^F-GE-180, confirming specificity of these tracers (Supplemental Fig. 3).

Quantification of spleen uptake revealed significant increases for both tracers from 2 to 6 d after stroke (^11^C-DPA-713: 7.68 ± 1.84 vs. 12.25 ± 3.65 %ID/g; ^18^F-GE-180: 12.57 ± 1.11 vs. 14.16 ± 3.12 %ID/g) (Supplemental Fig. 5). Additionally, a significant increase was observed between 6 and 28 d with ^18^F-GE-180 (14.16 ± 3.12 vs. 20.19 ± 4.3 %ID/g) but not with ^11^C-DPA-713. No correlation was observed between tracer uptake in the spleen and the infarct ROI.

**FIGURE 4.**
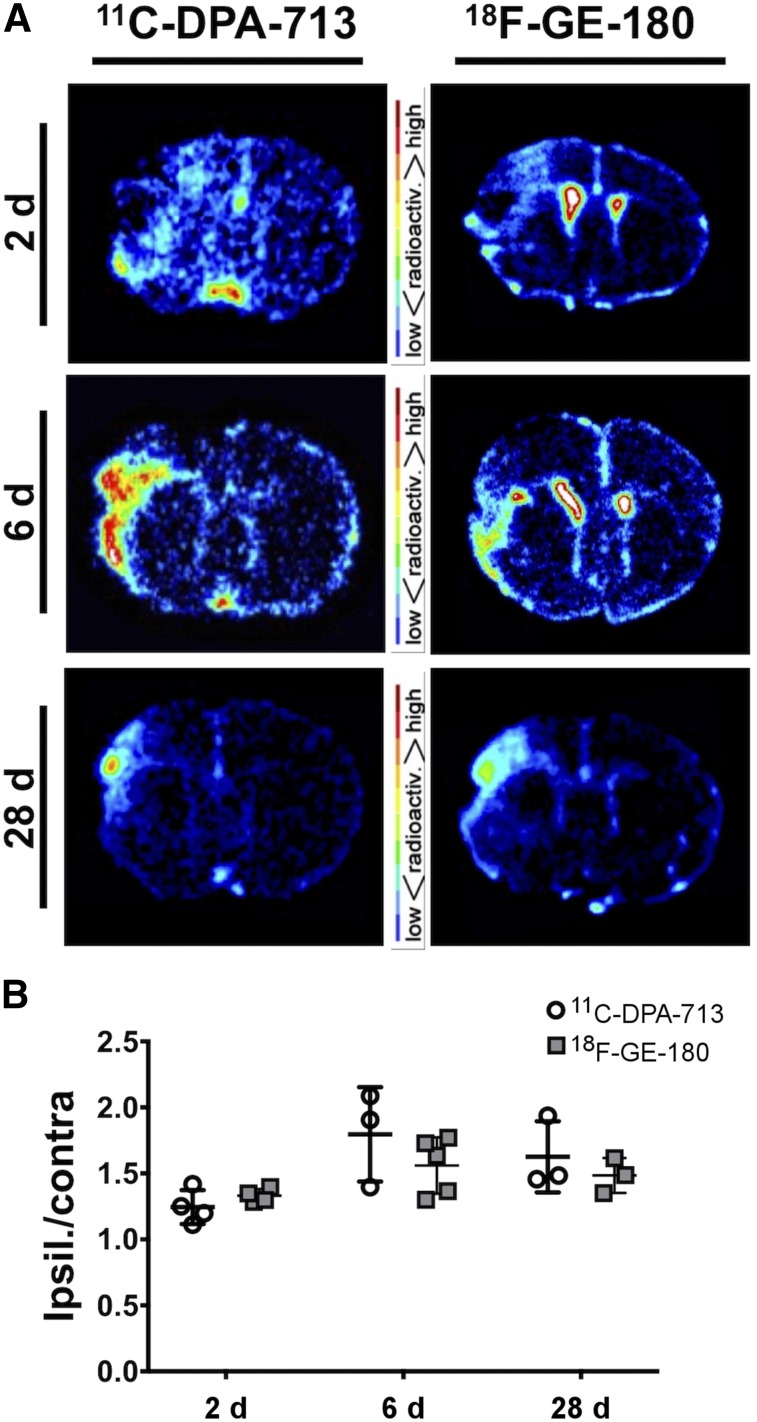
(A) Representative ex vivo coronal brain autoradiography images of ^11^C-DPA-713 and ^18^F-GE-180 uptake at 2, 6, and 28 d after dMCAO surgery. (B) Ratios of mean pixel intensity in ipsilateral versus contralateral brain ROIs for ^11^C-DPA-713 and ^18^F-GE-180 autoradiography (mean ± SD).

Ex vivo digital autoradiography results support in vivo PET findings with increased tracer uptake seen in the ipsilateral compared with contralateral hemisphere (Fig. 4A). Ipsilateral-to-contralateral ratios were greater than 1 for both tracers at all time points, indicating increased binding in infarcted tissue (Fig. 4B). In line with in vivo findings, ipsilateral-to-contralateral ratios did not differ significantly between tracers at any time point.

**FIGURE 5.**
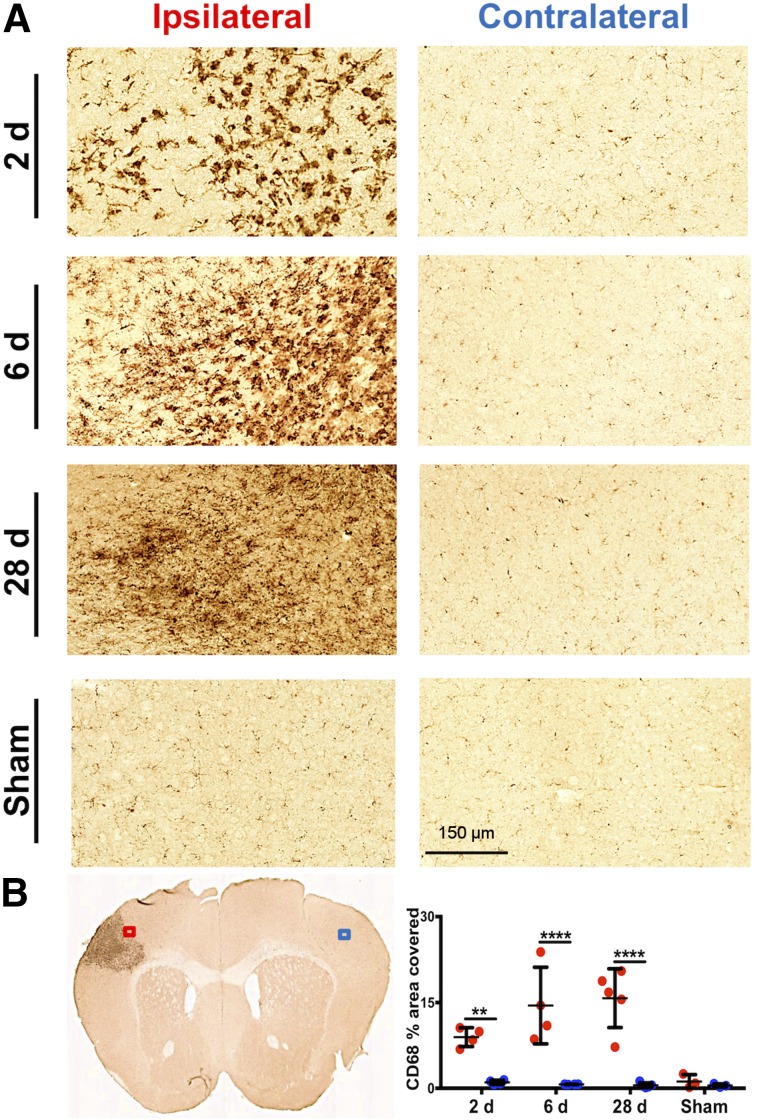
(A) Representative images of CD68 immunostaining in ipsilateral infarct border versus contralateral brain tissue after dMCAO and sham surgery (20× magnification). (B) Representative 6-d dMCAO brain section and quantification of percentage area covered with CD68 staining (mean ± SD) in ipsilateral (ROI indicated by red box) and contralateral (ROI indicated by blue box) tissue of dMCAO and sham animals. Two-way ANOVA, Sidak’s post hoc tests (**P < 0.01, ****P < 0.0001).

**FIGURE 6.**
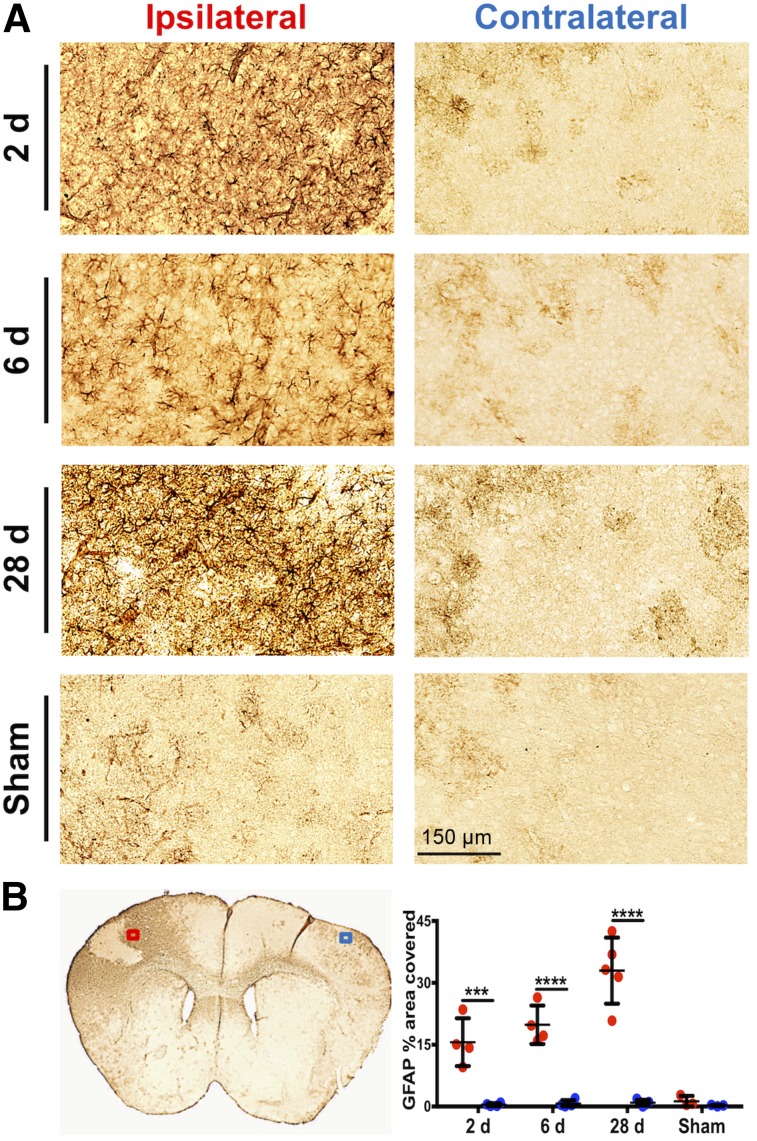
(A) Representative images of GFAP immunostaining in ipsilateral infarct border versus contralateral brain tissue after dMCAO and sham surgery (20× magnification). (B) Representative 6-d dMCAO brain section and quantitation of percentage area covered with GFAP staining (mean ± SD) in ipsilateral (ROI indicated by red box) and contralateral (ROI indicated by blue box) tissue of dMCAO and sham animals. Two-way ANOVA, Sidak’s post hoc tests (***P < 0.001, ****P < 0.0001).

**FIGURE 7.**
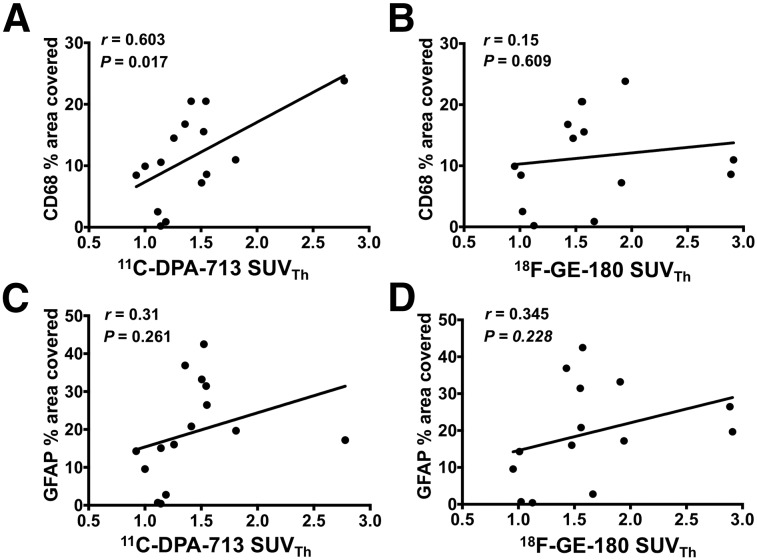
Correlation between in vivo ^11^C-DPA-713 and ^18^F-GE-180 signal (SUV_Th_) and ex vivo CD68 (microglia/macrophage) and GFAP (astrocyte) immunostaining across all groups (^11^C-DPA-713: day 2, *n* = 3; day 6, *n* = 4; day 28, *n* = 4; sham, *n* = 3; and ^18^F-GE-180: day 2, *n* = 2; day 6 *n* = 4; day 28, *n* = 4; sham, *n* = 3). Pearson correlation.

CD68 immunostaining of brain tissue, reflecting activated microglia/macrophages, corresponded well with PET and autoradiography results (Fig. 5). Quantification revealed markedly elevated levels of CD68 staining in the ipsilateral (infarct border) compared with contralateral brain tissue of dMCAO mice (Fig. 5B; 2 d: 8.95 ± 1.65 vs. 1.05 ± 0.37, *P* = 0.0084, *n* = 4; 6 d: 14.47 ± 6.69 vs. 0.69 ± 0.03, *P* < 0.0001, *n* = 4; 28 d: 15.76 ± 5.13 vs. 0.54 ± 0.42, *P* < 0.0001, *n* = 5), whereas low levels of staining were observed in 6-d sham mice (1.20 ± 1.19 vs. 0.50 ± 0.29, *P* = 0.998, *n* = 3). Similarly, GFAP levels were significantly elevated in ipsilateral versus contralateral tissue of dMCAO (Fig. 6; 2 d: 15.61 ± 5.80 vs. 0.46 ± 0.37, *P* = 0.0001, *n* = 4; 6 d: 19.83 ± 4.67 vs. 0.71 ± 0.87, *P* < 0.0001, *n* = 4; 28 d: 32.97 ± 8.00 vs. 0.25 ± 0.16, *P* < 0.0001, *n* = 5) but not sham mice. In vivo ^11^C-DPA-713 PET signal significantly correlated with ex vivo CD68 levels (Fig. 7A, *r* = 0.603, *P* = 0.017, *n* = 15), whereas no significant correlation was observed with ^18^F-GE-180 (Fig. 7B). GFAP levels did not correlate with the in vivo TSPO PET signal from either tracer (Figs. 7C and 7D). These results were supported by colocalization of TSPO and CD68 expression in the infarct of 6-d dMCAO mice. TSPO immunofluorescence staining was not observed on GFAP-positive cells (Supplemental Fig. 6).

## DISCUSSION

Here, we report the first, to our knowledge, head-to-head comparison of 2 promising second-generation TSPO tracers, ^11^C-DPA-713 and ^18^F-GE-180, in a rodent model of cerebral ischemia. The overall goal was to evaluate the sensitivity and accuracy of these tracers at both acute and chronic time points after dMCAO surgery.

Here, we prove TSPO PET is a valuable tool for detecting acute and chronic neuroinflammation in cerebral ischemia. ^11^C-DPA-713 appeared to be more sensitive than ^18^F-GE-180, with successful delineation of neuroinflammation in the infarcted ROI at 2 d after dMCAO, when ^18^F-GE-180 did not detect a significant difference. These results are consistent with previous reports demonstrating the high sensitivity of ^11^C-DPA-713 to detect subtle inflammatory changes, with a recent study demonstrating significant increases in ^11^C-DPA-713 uptake with normal aging that was not evident with ^11^C-PK11195 and has previously been only identifiable via ex vivo means. Both tracers effectively identified inflammation in the ipsilateral ROI at 6 and 28 d after dMCAO, highlighting the potential of TSPO PET for quantifying and tracking chronic inflammation poststroke. Similar results were found using SUV_Cb_, indicating the translational potential of these tracers for imaging stroke patients. Additionally, preblocking with cold PK11195 revealed significantly decreased binding of both tracers in dMCAO mice at 6 d after stroke, confirming the specificity of both tracers for TSPO.

In vivo PET results were validated by ex vivo autoradiography and immunostaining of activated microglia/macrophages and astrocytes. Autoradiography confirmed increased tracer uptake in infarcted tissue for both tracers at all time points. Ipsilateral-to-contralateral ratios for ^18^F-GE-180 and ^11^C-DPA-713 obtained with autoradiography were slightly different from those observed with PET. Notably, a slightly higher ratio was seen with ^18^F-GE-180 autoradiography at 2 d. A possible explanation for the lack of discrimination using in vivo ^18^F-GE-180 PET at this time point may be the limited resolution of the PET scanner to detect this difference or the increased blood pool concentration of ^18^F-GE-180 resulting in reduced signal to background, masking differential uptake.

Increased ipsilateral TSPO PET signal was supported by striking increases in CD68 immunostaining in the ipsilateral infarct border. Moreover, correlation of in vivo PET and ex vivo immunostaining of tissues from the same animals revealed novel insights into the comparative specificity of these tracers. ^11^C-DPA-713 PET signal correlated significantly with ex vivo levels of activated microglia/macrophages. Surprisingly, this was not observed with ^18^F-GE-180, indicating that ^11^C-DPA-713 PET more accurately depicts microglial activation. No correlation was found between TSPO PET and GFAP expression, suggesting that in the context of stroke, the TSPO PET signal mainly represents activated microglia/macrophages. This is in line with our findings demonstrating TSPO expression in the infarct core and border colocalizes almost exclusively with CD68, and agrees with results from a previous study using MCAO rats (28).

Our findings are consistent with previous reports in different rodent models of stroke demonstrating increased ipsilateral uptake using TSPO PET (19,21,25–28). However, few head-to-head studies have been conducted to date, and none have compared 2 second-generation TSPO tracers in stroke models. Enhanced sensitivity has previously been demonstrated with ^18^F-DPA-714 (19) and ^18^F-GE-180 versus ^11^C-PK11195 using the MCAO rat model (21). Additionally, ^18^F-DPA-714 has been shown to perform better in vivo, displaying a higher ipsilateral-to-contralateral ratio than both ^11^C-PK11195 and ^11^C-DPA-713 in a rat model of AMPA-induced unilateral neuroinflammation (20). Yet, increased ipsilateral-to-contralateral ratios were observed with ^18^F-GE-180 PET when compared with ^11^C-PK11195, which were not evident with ^18^F-DPA-714 using a similar model of unilateral neuroinflammation (22). However, ^18^F-GE-180 and ^18^F-DPA-714 imaging in these studies was conducted using separate cohorts and therefore did not directly compare tracers. The discrepancies between these studies highlights the need for direct head-to-head comparison studies in reproducible models of neuroinflammation, and emphasizes the importance of the current study, which will help guide future experimental design for investigating neuroinflammation after stroke.

The current work reveals novel insights into the temporal dynamics of neuroinflammation after stroke. Previous studies have reported increased TSPO PET binding in ROIs encompassing infarcted tissue ranging from 3 to 21 d after insult (19,21,25–28). However, these studies did not detect increased ipsilateral inflammation as early as 2 d after stroke, as demonstrated here with ^11^C-DPA-713. Moreover, few studies have investigated the effect of chronic inflammation after stroke. A study by Walberer et al., using ^11^C-PK11195 in a rat model of ischemia, demonstrated the highly dynamic process of inflammation with peak ^11^C-PK11195 binding occurring in the infarct at 7 d and spreading to thalamic regions detectable as far as 7 mo after stroke (33). Similarly, Walter et al. found that neuroinflammation spread to surrounding subcortical areas at 4 and 8 wk after permanent MCAO using ^11^C-PK11195 (34). These results indicate a local versus remote microglial activation phenomena that warrants further investigation using more sensitive tracers.

Since the spleen is central to the activation, proliferation, and trafficking of immune cells to the site of injury in the early stages after stroke (35), we quantified spleen PET signal and found a stepwise increase in TSPO binding after dMCAO. Although this increase did not correlate with brain inflammation, it highlights the importance of the dynamic changes occurring in the periphery after stroke and demonstrates the benefits of using full-body TPSO PET to investigate these processes. To our knowledge, this is the first investigation of peripheral TSPO PET in stroke.

## CONCLUSION

Here, we present the first head-to-head comparison of 2 second-generation TSPO PET tracers in a rodent model of ischemic stroke. Both tracers enabled identification of acute and chronic inflammation in infarcted brain tissue; however, ^11^C-DPA-713 PET more accurately reflected microglial/macrophage activation and afforded earlier detection than ^18^F-GE-180. Results from this study highlight the utility of TSPO PET as an invaluable tool for deciphering the in vivo role of neuroinflammation in both early and late stages after stroke in the central nervous system and periphery. Future work will focus on investigating the relationship between TSPO PET signal, neurologic symptoms, and long-term outcomes in rodent models and stroke patients.

## DISCLOSURE

This project was funded partly by a “Big Ideas in Neuroscience” grant from the Stanford Neurosciences Institute to the Stroke Collaborative Action Network (Buckwalter). No other potential conflict of interest relevant to this article was reported.
