# Supplementary material for: 11C-DPA-713 Versus 18F-GE-180: A Preclinical Comparison of Translocator Protein 18 kDa PET Tracers to Visualize Acute and Chronic Neuroinflammation in a Mouse Model of Ischemic Stroke
Source: J Nucl Med. 2019 Jan;60(1):122–8. doi: 10.2967/jnumed.118.209155 (PMC6354224; doi:10.2967/jnumed.118.209155)
Supplement: Supplementary file 1 [file jnm209155SupplementalData.pdf]

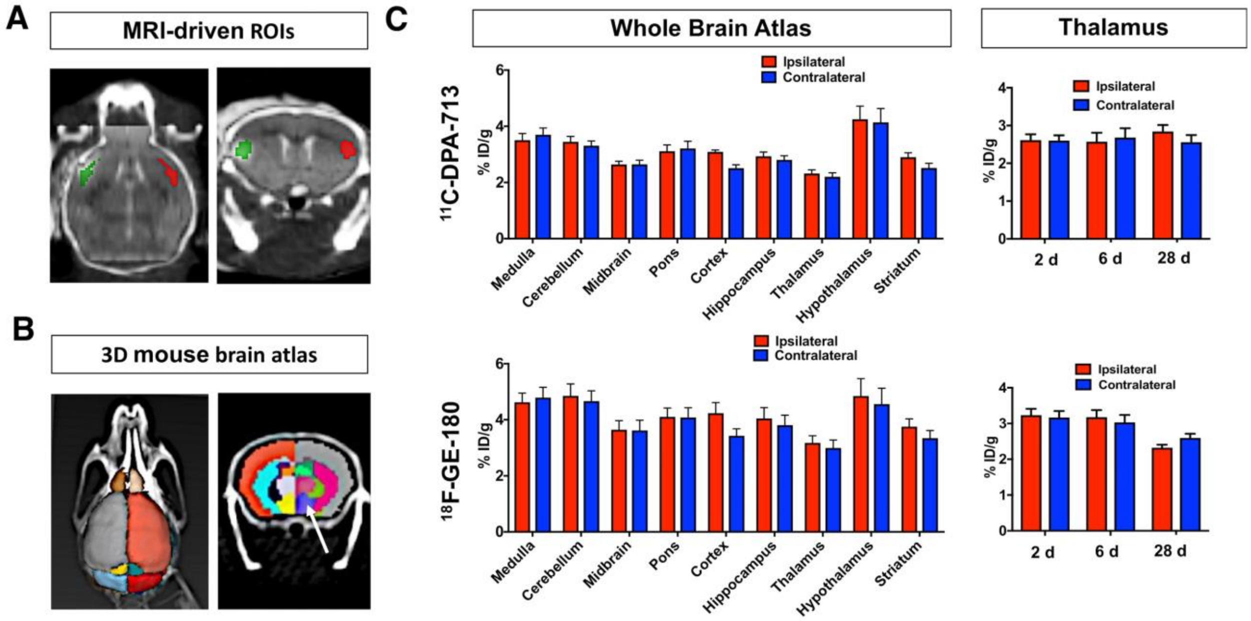

Supplementary Figure 1. MRI-driven ROI analysis (A). 3D mouse brain atlas analysis (B). Results from whole brain atlas revealed the contralateral thalamus as a suitable pseudo-reference region (C).

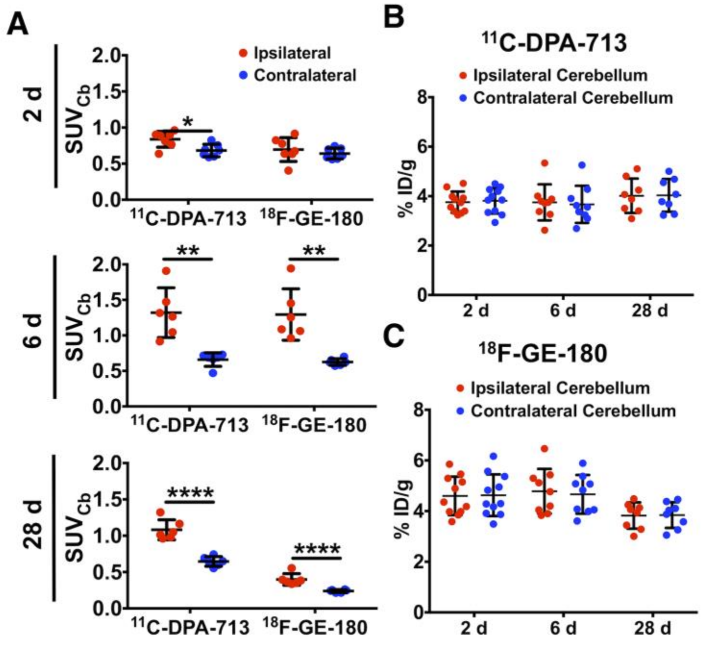

Supplemental Figure 2. PET image quantification of ^{11}C-DPA-713 and ^{18}F-GE-180 uptake in dMCAO mice using the ipsilateral cerebellum (SUV_{Cb}) (A). ^{11}C-DPA-713 (B) and ^{18}F-GE-180 (C) uptake in the ipsilateral and contralateral cerebellum did not differ in dMCAO mice over time.

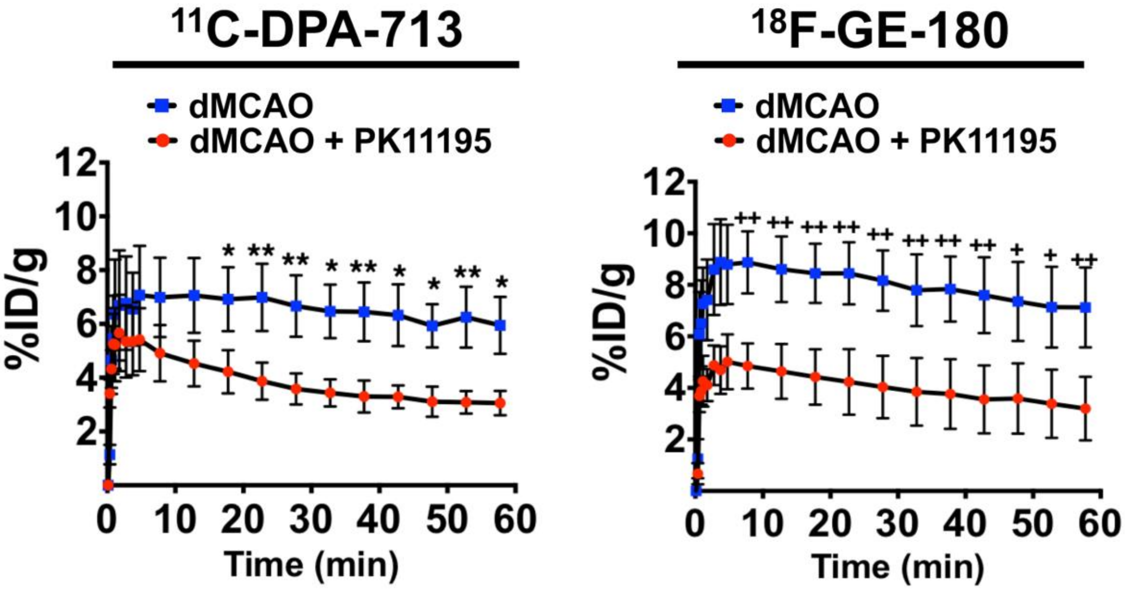

Supplemental Figure 3. Time activity curves depicting tracer uptake in the infarct of dMCAO mice with and without pre-blocking with PK11195 (3mg/kg) at 6 days post-stroke (mean ± SD). Wilcoxon matched paired test (*p<0.05, **p<0.01, +p<0.001, ++p<0.0001).

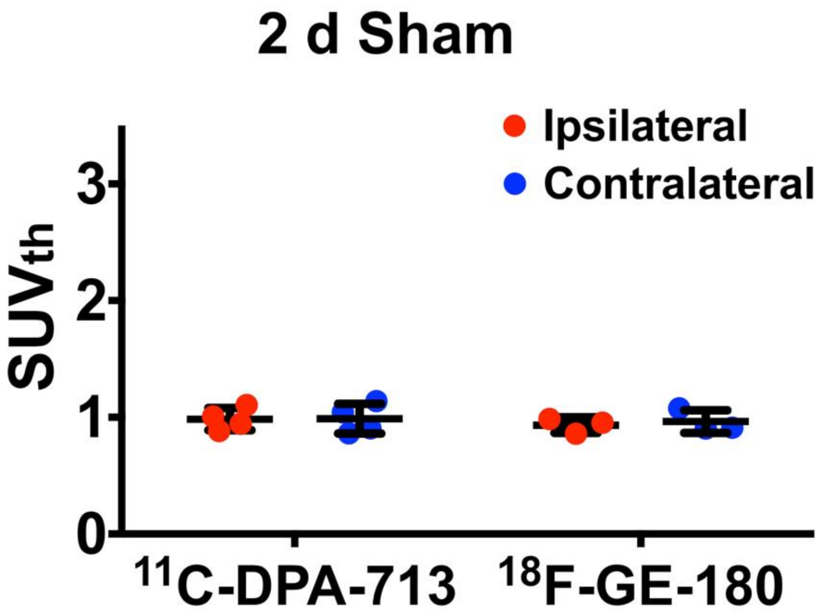

Supplemental Figure 4. ^{11}C-DPA-713 and ^{18}F-GE-180 uptake (SUV_{Th}) in ipsilateral and contralateral ROIs in sham mice 2 days post-surgery (mean ± SD).

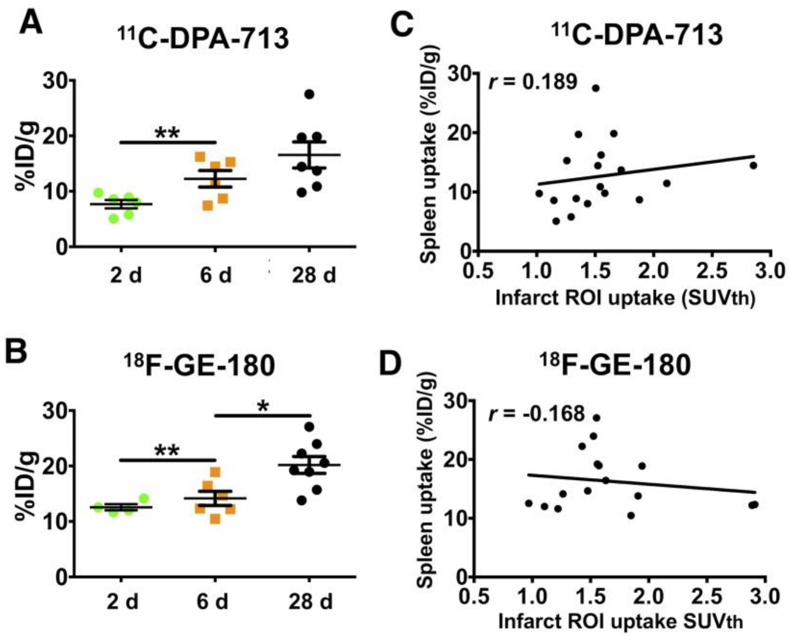

Supplemental Figure 5. ^{11}C-DPA-713 and ^{18}F-GE-180 spleen uptake (%ID/g) (A, B). Relationship between spleen uptake (%ID/g) and infarct uptake (SUV_{Th}) using Pearson's correlation for ^{11}C-DPA-713 (C) and ^{18}F-GE-180 (D).

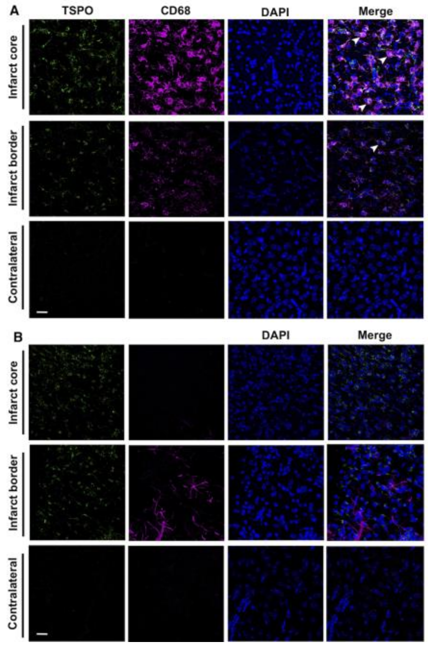

Supplemental Figure 6. Immunofluorescent TSPO/CD68 (A) and TSPO/GFAP (B) double staining depicting that expression of TSPO is primarily in CD68-positive but not GFAP-positive cells at 6 days post-dMCAO.
